# Hormone-replacement therapy influences gene expression profiles and is associated with breast-cancer prognosis: a cohort study

**DOI:** 10.1186/1741-7015-4-16

**Published:** 2006-06-30

**Authors:** Per Hall, Alexander Ploner, Judith Bjöhle, Fei Huang, Chin-Yo Lin, Edison T Liu, Lance D Miller, Hans Nordgren, Yudi Pawitan, Peter Shaw, Lambert Skoog, Johanna Smeds, Sara Wedrén, John Öhd, Jonas Bergh

**Affiliations:** 1 Department of Medical Epidemiology and Biostatistics, Karolinska Institutet, Stockholm, Sweden; 2 Department of Oncology and Pathology, Cancer Center Karolinska, Karolinska Institutet and Hospital, Stockholm, Sweden; 3 Bristol-Myers Squibb, Princeton, NJ, USA; 4 Genome Institute of Singapore, Republic of Singapore; 5 Department of Pathology, Uppsala University Hospital, Uppsala, Sweden

## Abstract

**Background:**

Postmenopausal hormone-replacement therapy (HRT) increases breast-cancer risk. The influence of HRT on the biology of the primary tumor, however, is not well understood.

**Methods:**

We obtained breast-cancer gene expression profiles using Affymetrix human genome U133A arrays. We examined the relationship between HRT-regulated gene profiles, tumor characteristics, and recurrence-free survival in 72 postmenopausal women.

**Results:**

HRT use in patients with estrogen receptor (ER) protein positive tumors (n = 72) was associated with an altered regulation of 276 genes. Expression profiles based on these genes clustered ER-positive tumors into two molecular subclasses, one of which was associated with HRT use and had significantly better recurrence free survival despite lower ER levels. A comparison with external data suggested that gene regulation in tumors associated with HRT was negatively correlated with gene regulation induced by short-term estrogen exposure, but positively correlated with the effect of tamoxifen.

**Conclusion:**

Our findings suggest that post-menopausal HRT use is associated with a distinct gene expression profile related to better recurrence-free survival and lower ER protein levels. Tentatively, HRT-associated gene expression in tumors resembles the effect of tamoxifen exposure on MCF-7 cells.

## Background

There is convincing evidence that users of HRT are at increased risk of breast cancer, that risk increases with duration of use, and that the risk is substantially greater for combined estrogen-progestin than for estrogen-only HRT [1-4]. The impact of HRT use on breast-cancer prognosis and clinical characteristics is, however, not well understood. Results from a large randomized clinical trial, the Women's Health Initiative, indicate a poorer outlook in users of combined estrogen-progestin therapy, represented by a larger proportion of tumors with lymph-node metastases and by differentiated tumors [3,4]. In contrast, observational studies have repeatedly reported less malignant clinical features as well as improved prognosis in HRT users [5-8]. This finding may be due to biases such as closer medical surveillance and reduced sensitivity and specificity of mammography screening in women on HRT, and the exclusion of women with preclinical breast-cancer lesions before initiation of hormone therapy [5,8].

The effect of estrogen is mediated through its receptors in concert with co-activators and co-repressors [9-12]. Through transcriptional mechanisms involving the ER, estrogens regulate proliferation and cell cycle progression. In addition, estrogens have also been postulated to influence the regulation of cell death and genomic instability of cells [12].

Expression microarrays have been employed in the analysis of breast cancers and can provide better prognostic information compared with standard clinical and pathological parameters [13-17]. Microarray analyses appear to be able to discriminate sporadic versus hereditary breast cancer [18] and to identify array profiles that are strongly associated with ER status [13,14,16]. *In vitro *studies have confirmed consistent effects of exogenous estrogens on gene expression in human cell lines [19,20] and in animals [21].

In this study, we aimed to compare gene expression of breast cancers in HRT users and non-users and to correlate the expression pattern to recurrence-free survival. We further investigated whether the gene expression pattern-survival relation would hold in an independent cohort of patients with breast cancer. Finally, we explored the possible mechanism behind such a link by comparison with external gene-expression data from an estrogen- and tamoxifen-treated cell line.

## Methods

### Study populations

All women (n = 524) operated on for breast cancer at Karolinska Hospital, Stockholm, Sweden, from 1 January 1994 through 31 December 1996, were included in the study. Patients were excluded because of: lack of frozen tumor tissue (n = 231), insufficient quality of tumor material (n = 103), actively refusing consent (n = 6), or emigration (n = 7). Another 18 patients were excluded for clinical reasons (neoadjuvant chemotherapy, in situ cancer, or recurrence after surgery within 1 month). Of the 159 remaining patients, 98 were postmenopausal. We obtained information on use of HRT and other breast-cancer risk factors from the case records. Users were defined as patients on HRT at time of referral to the Karolinska Hospital. Non-users were those actively stating no current or previous use of HRT. Ten patients who were former users of HRT were excluded, leaving 88 patients in the study, of which 32 received HRT at time of diagnosis and 56 were non-users. Of the 32 users, 20 used a combined estrogen and progesterone regimen, 11 used estrogen only, and for one patient, detailed information was unavailable. The mean duration of use was 7.2 years and 38% had used hormones for more than 5 years. The patients were followed for at least 8 years after diagnosis, until time of recurrence or death, whichever event occurred first. This cohort will be referred to as the "study cohort".

Data from 131 postmenopausal women surgically treated for ER-positive breast cancer in 1987–89 at the University Hospital of Uppsala, Sweden were used for validation [22]. Gene expression data were obtained in the same way as for the study cohort. Hereafter we refer to this cohort as the "validation cohort". Twelve-year follow-up data, clinical stage, Elston grade, ER and progesterone receptor (PR) status, and information about adjuvant hormonal therapy was available for these patients, but there was no information about HRT use.

The institutional review boards at Karolinska Institutet and Hospital, respectively, approved the microarray expression studies.

### Molecular analyses 

ER and progesterone receptor (PR) status was measured in cytosol by enzyme immunoassay [22]. Values above 0.5 fmol/g of cytosol protein were considered to indicate positive receptor-protein expression. For some analyses, we used the absolute measurement of the receptor protein, measured in fmol/g of cytosol protein. Elston grade was blindly reassessed by one of the authors (HN) using predefined criteria [23].

RNA was prepared using RNeasy spin column kit (Qiagen, Valencia, CA, USA). Frozen tumors were cut into minute pieces and homogenized for 40 seconds in RNeasy Lysis Buffer (RLT). Proteinase K was added, [24] and the samples were incubated for 10 minutes at 55°C, followed by centrifugation and the addition of ethanol. After the transfer onto RNeasy columns, DNase was added to increase RNA quality. RNA quality was assessed using an Agilent 2100 bioanalyzer (Agilent Technologies, Rockville, MD). The material was stored at -70°C. The amount of RNA for each probe preparation varied between 2 and 5 μg. First-strand cDNA synthesis was generated by using a T7-linked oligo-dT primer, followed by second-strand synthesis. The in vitro transcription reactions were performed in batches to generate biotinylated cRNA targets, which were subsequently chemically fragmented at 95°C for 35 minutes.

Ten μg of the fragmented, biotinylated cRNA was hybridised at 45°C for 16 hours to an Affymetrix high-density oligonucleotide array (human genome U133 A Genchip^®)^. These were then washed and stained with streptavidin-phycoerythrin (10 μg/ml). Signal amplification was achieved using a biotinylated anti-streptavidin antibody. The scanned images were inspected for the presence of artifacts. In case of defects, the hybridisation procedure was repeated. Expression values and detection calls were computed from raw data following the procedures outlined for the Affymetrix MAS 5.0 analysis software. Global mean normalization of the MAS 5.0 expression was used to reduce differences in chip intensity [25].

A sample was either re-labeled, and the hybridization repeated, or excluded from further analysis if a scaling factor >4 was necessary, <30% present calls were found, or the squared multiple correlation coefficient of the expression data on the array to all other arrays was <0.6 [26].

Expression data for 22,283 probe sets and 88 breast primary tumors were used. Probe sets which were not detected in at least 50% of the tumors were excluded, which led to a final 11,295 probe sets, representing 8113 genes for analysis. The expression data has been deposited at the GEO repository under the accession number GSE1456.

### Comparisons with cell-line experiments

In the cell-line experiments by Finlin et al [27], estrogen-deprived MCF-7 cells were cultured in 10^-8 ^M β-17-estradiol for 4, 8, and 24 h. The tamoxifen-treated samples were exposed to either 1 or 6 μM of tamoxifen for 48 h.

### Statistical analyses

We computed gene-wise test statistics t_i _to measure the strength of the relationship between the expression of gene g_i _and the clinical variable of interest. For the association with HRT use, we calculated the two-sample Welch t statistic. For association with age, we used the Spearman rank correlation coefficient. Larger absolute values of either statistic correspond to a stronger association with HRT status and age, respectively. Positive values of the statistic indicate that a gene is over-expressed in HRT users, and *vice versa*.

The cut-off for deciding that a gene g_i _is significantly differentially expressed is not based on the usual p values. Instead, we select genes based on their local false discovery rate (FDR) as described in [26]. The quantity fdr_i _associated with gene g_i _specifies the proportion of false-positive results that can be expected among genes that have the same test statistic t_i_. Equivalently, we can interpret 1 minus fdr_i _as the probability that gene g_i _is truly associated with the clinical variable of interest. The genes under study were assumed to be a mixture of differentially and non-differentially expressed genes, where the distribution of t statistics for non-differentially expressed genes can be inferred from random permutations of the data.

Initially, all genes with local FDR <0.2 were selected as candidate genes for discriminating between HRT users and non-users. This liberal cut-off point was chosen because the aim of the present study was not to identify specific genes, but rather patterns of expression. A high cut-off level for gene selection would be more likely to identify genes strongly linked to exposure to HRT, but not comprehensive biologically relevant patterns.

Preliminary analysis of the clinical data indicated that HRT use was associated with age, and thus that age is a possible confounding factor for the relationship between gene expression and HRT use (Table 1). We therefore removed HRT-associated genes that were significantly correlated with age in the reference group of ER-positive non-HRT users (n = 50). The significance cut-off point was again based on the local FDR estimate, here for the Spearman rank correlation coefficient. Genes with local FDR <0.4 for age were excluded from the list of HRT candidate genes.

The 72 patients with ER-positive tumors in the study cohort were hierarchically clustered based on the age-adjusted HRT candidate gene list. We used average linkage and Euclidean distances; each gene was robustly standardized by subtracting the median and dividing by the interquartile range of expression prior to clustering. We used consensus clustering to assess the stability of the resulting grouping of samples and found it to be robust (see additional file 1).

We used Kaplan-Meier estimates with the log-rank test for establishing the univariate association between cluster membership and recurrence-free survival. We modeled multivariate survival using the Cox proportional hazards model. Hazard ratios and their 95% confidence intervals were computed from the Wald test statistics; the p value for each variable is based on the likelihood ratio test comparing the full model and the model without the tested variable, using the asymptotic χ^2 ^distribution of the likelihood ratio. The multivariate models were adjusted for age, Elston grade, tumor size, lymph node status, PR status, and HRT use.

For validation, we used a simple supervised clustering scheme, in which groups identified in the study cohort through hierarchical clustering were characterized via their centroids (i.e. by averaging gene expression for each group). All patients in the validation cohort were assigned to the centroid with the smallest Euclidean distance for the same robustly standardized genes.

For comparison with MCF-7 cells, we chose genes that were found to be responsive to both estrogen and tamoxifen, based on the experimental data described previously and deposited in the Stanford Microarray Database [27]. Cells were treated with either 10 nM 17β-estradiol and harvested at 4, 8, and 24 hours following treatment, or with 1 and 6 μM of tamoxifen and harvested after 48 hours. Genes chosen showed a consistent minimum fold change for estradiol exposure and an inverse fold change for tamoxifen exposure, when compared with untreated control groups (see additional file 1). Response to hormone treatment and sensitivity to tamoxifen is a standard criterion for defining ER-regulated genes, and while it will not capture all anti-estrogen-responsive genes [28], we were specifically interested in genes that can be expected to be regulated by the presence of both HRT and tamoxifen. For genes that fulfilled this criterion and were associated with HRT use, we computed fold changes between HRT users and non-users, and compared them with the fold changes found in the experimental data.

## Results

Non-HRT users were older than HRT users at time of diagnosis (p = 0.001; Table 1), but no difference in average tumor size (p = 0.39) or proportion of patients with lymph-node metastasis (p = 1.0) was evident. Significantly more ER negative tumors were seen in HRT users (31% versus 11%, p = 0.02). No difference in progesterone-receptor status, recurrence-free survival (defined as lack of distant metastases or death within 5 years of diagnosis) or Elston grade was seen between users and non-users of HRT (Table 1). Comparisons using only patients with ER-positive tumors yielded similar results.

A preliminary study of the distribution of t statistics comparing gene expression between HRT users and non-users for all 88 patients indicated that the observed differences are almost exclusively due to the differences observed in the ER-positive tumors, whereas the ER-negative tumors contribute little or no information on differential expression (see supplementary Figure 1 and the associated discussion in additional file 1). As the difference in expression profiles between HRT-users and non-users was confined to ER-positive tumors, we limited all further analysis to this group (n = 72).

Among the 72 patients with ER-positive tumors, 22 were HRT users and 3 (14%) experienced recurrence or death due to breast cancer within 5 years of diagnosis. Recurrence among non-users was seen in 14 patients (28%; p = 0.24).

We observed 331 genes to be differentially expressed between HRT users and non-users, with a local FDR ≤ 0.2, of which 54% were down-regulated in HRT users. We also found 580 genes that were correlated with age in non-users of HRT with a local FDR ≤ 0.4. We removed the 55 genes that were on both lists, which yielded an age-adjusted HRT associated gene list of 276 genes (The annotated list of genes can be found in additional file 2).

### Molecular classification of patients based on HRT- associated genes

**Figure 1:**
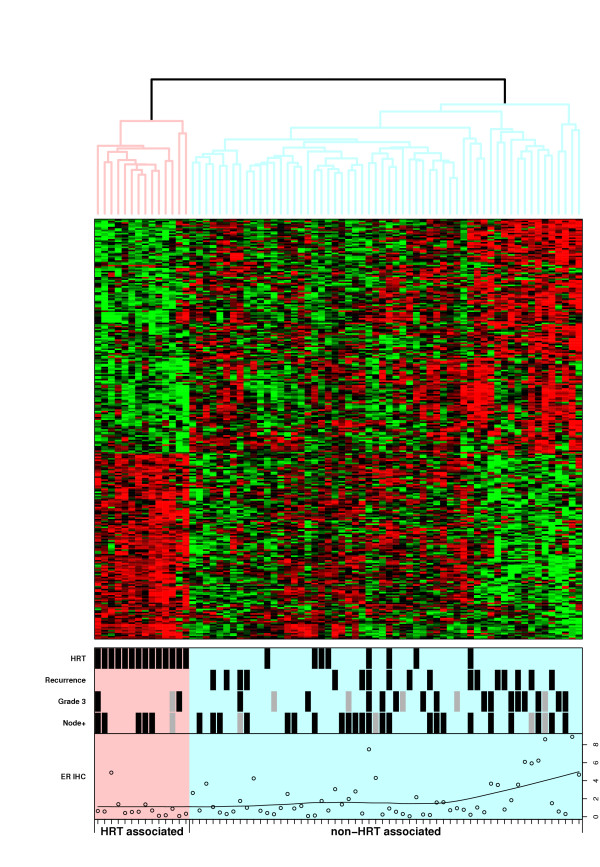
**Unsupervised clustering of the study cohort**. Hierarchical clustering using 276 HRT associated genes in ER positive tumors (n = 72). Each column corresponds to a patient, each row to a gene; expression level is indicated on a color scheme from green/low to red/high. Patients are labeled according to HRT use, recurrence-free survival (= recurrence, defined as distant metastases or death within 5 years of diagnosis), Elston grade 3, lymph node status, and ER level measured on the protein level. Gray symbols indicate missing values.

The 72 ER-positive cancers were grouped using hierarchical clustering on the expression profiles of the 276 genes (Figure 1). Each column corresponds to a patient labeled according to HRT use, recurrence or death within 5 years, Elston grade 3, lymph node metastasis, and ER protein levels. Two distinct clusters emerged. Obviously, these clusters differed significantly in the proportion of patients receiving HRT (100% in the HRT-associated cluster versus 13% in the non-HRT-associated cluster, p < 10E-9). There was, however, no significant difference with regard to type and duration of HRT use between the groups. In the HRT-associated cluster, 11 (77%) out of 13 patients used combined therapy with estrogen and progesterone, and the average duration of HRT use was 6.2 years. The corresponding figures in the non-HRT associated cluster were 4 (50%) out of 8 HRT users (p = 0.34) and 8.8 years (p = 0.42), respectively.

The clusters differed significantly with regard to ER protein levels (p = 0.02), but not Elston grade (p = 0.67) or lymph node status (p = 1.00). All patients in the HRT-associated cluster were recurrence free 5 years after diagnosis and thus, had a significantly better recurrence-free survival (p = 0.03), even after adjusting for Elston grade, tumour size, lymph node status, PR status, HRT use, and age in the multivariate model (p = 0.001, see supplementary Table 1 in additional file 1).

### Validation of molecular classification

**Figure 2:**
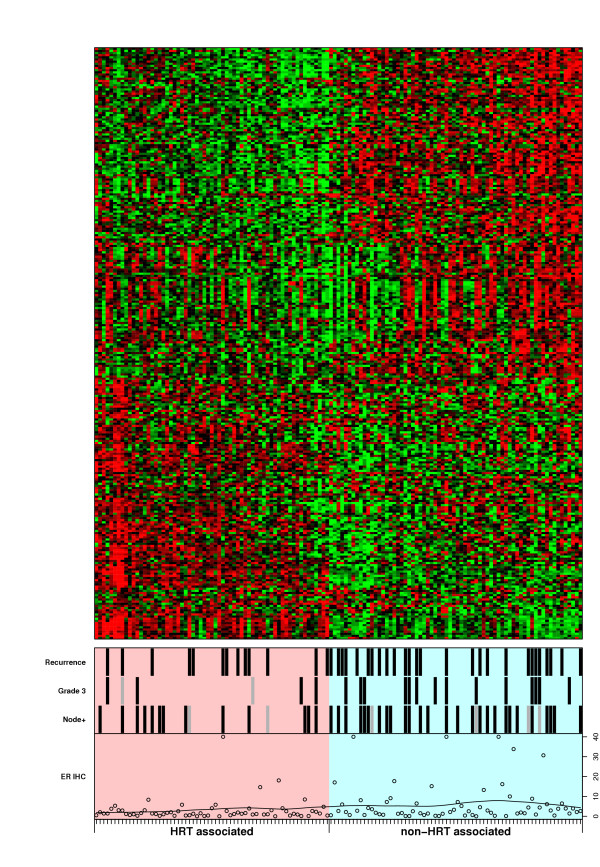
**Supervised clustering of the validation cohort**. Supervised clustering of 131 ER-positive postmenopausal Uppsala patients using the 276 genes identified in the Stockholm material, shown by row and in the same order as in Figure 1. Each column corresponds to a patient labeled according to survival (= recurrence; defined as distant metastases or death within 5 years of diagnosis), Elston grade 3, and lymph node status, where gray symbols indicate missing values. ER protein values were truncated at 40 for display purposes.

In the validation cohort, the clustering split the 131 tumors into two groups of 68 non-HRT-like and 63 HRT-like expression profiles (Figure 2). Each column corresponds to a patient labeled according to recurrence or death within 5 years, Elston grade 3, lymph-node metastasis, and ER protein levels. As no HRT information was available for the validation cohort, the data could only be used to confirm the prognostic potential of the clustering. Tumors with HRT-associated profiles had better recurrence-free survival (p = 0.02), lower Elston grade (p = 0.02), lower protein levels of ER (p = 0.02), and were more often node-negative. When only patients treated with adjuvant tamoxifen (n = 51) were included, we found patients in the HRT-associated cluster to have a significantly better survival (p = 0.04), even after adjusting for the effects of Elston grade, lymph node status, PR status, tumor size, and age at time of diagnosis in the multivariate model (p = 0.001, see supplementary Table 1 in additional file 1). For patients not receiving adjuvant hormonal therapy (n = 80), the the survival difference between the clusters was still significant (p = 0.03), even after adjustment for potential confounders (p = 0.02), but the relationship between the clusters was reversed; patients in the HRT-associated cluster did worse, indicating that tamoxifen influenced the apparent beneficial prognostic effect of HRT-associated gene expression. This effect was not seen in the study cohort, probably explained by the small number of patients (n = 7; 8%) left without adjuvant hormonal therapy.

**Figure 3:**
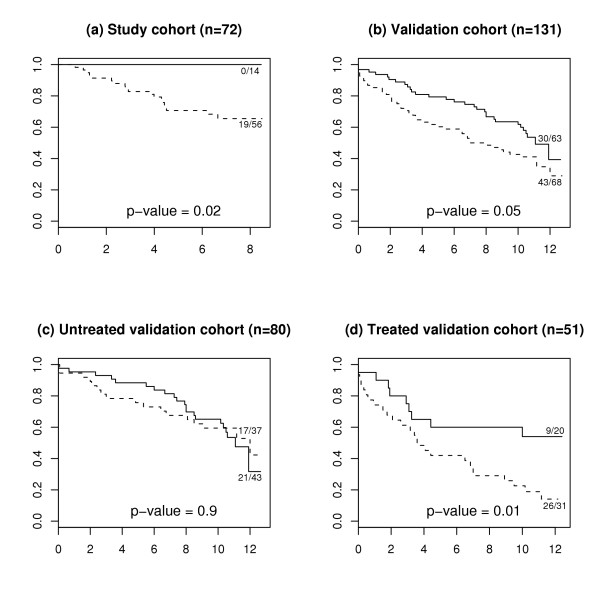
**Survival curves for study and validation cohort, by expression profile cluster**. Kaplan-Meier curves comparing recurrence-free survival (defined as no death and no distant metastasis) for patients with HRT-like expression profiles (solid line) and patients with non-HRT-like expression lines (dashed lines). Follow-up was 8 years for the study cohort and 12 years for the validation cohort. Number of events/number of patients are shown for each group next to the corresponding curve. The p values are calculated via the log-rank test. Panels (a) and (b) show the full study and validation cohorts, respectively, as described in the paper; the treated and untreated sub-cohorts of the validation cohort refer to adjuvant hormonal therapy, as all patients received surgery and radiotherapy.

Figure 3 shows the survival (Kaplan-Meier curves, i.e. no adjustment) of the patient cohorts with the respective gene expression signatures. While patients with the HRT-like signature did better in the overall validation cohort (Figure 3b), the main benefit was experienced by those receiving adjuvant hormonal therapy (Figure 3d), whereas the small benefit for those treated only with surgery and radiation therapy was convincingly non-significant (Figure 3c).

### Comparison of expression-fold changes with cell-line results

**Figure 4:**
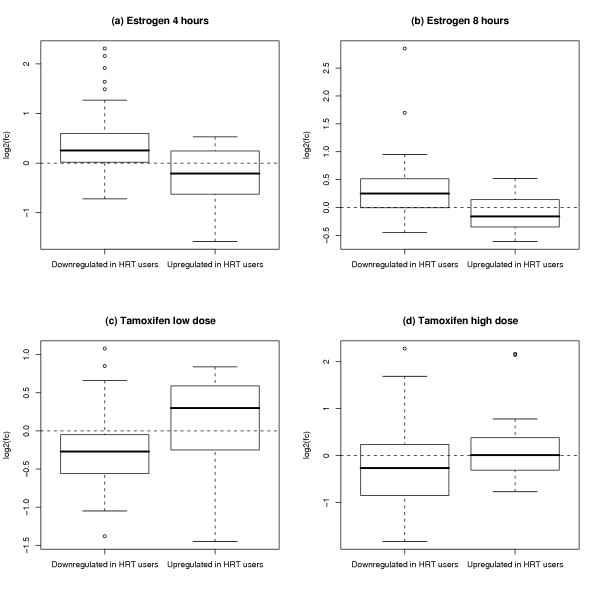
**Comparison with cell line experiments**. Fold changes induced by estrogen and tamoxifen treated MCF-7 cell lines for unique genes that are down-regulated (n = 54) or up-regulated (n = 30) by HRT in breast cancer patients. The fold changes are shown on the log_2 _scale, so the dashed line at zero corresponds to no differential expression, positive values correspond to up-regulation in treated cells, and negative values correspond to down-regulation in treated cells compared with untreated control cells (see additional file 1 on fold change calculation and gene matching).

Of the 276 genes associated with HRT use, 84 unique genes corresponding to 96 probe sets were found to be estrogen- and tamoxifen-responsive in the experimental data described in [27] (see additional file 3). In order to compare the regulatory effect of HRT in breast cancer, and estrogen and tamoxifen in cell lines, we grouped genes into those that are down-regulated (n = 54) and up-regulated (n = 30) in HRT users. Figure 4 summarizes the fold changes induced in these two groups of genes by estrogen and tamoxifen. Fold changes are shown on a log_2 _scale, so the dashed line at zero corresponds to no differential expression, whereas positive values indicate upregulation in treated cells and negative values indicate downregulation in treated cells compared with untreated controls. Figures 4a and 4b indicate a negative correlation between the effects of HRT and estrogen; the genes that are downregulated by HRT are predominantly upregulated by estrogen, and vice versa. In contrast, we saw positive correlation between the effects of HRT and low doses of tamoxifen (Figure 4c); Figure 4d shows a similar, although weaker relationship for high doses of tamoxifen.

## Discussion

We have shown that a gene expression profile in ER-positive HRT users, based on 276 genes, is significantly associated with better survival, despite being linked to lower levels of the ER protein. HRT use by itself could not be used as a predictor of outcome. The gene-expression profile remained significantly associated with survival even after adjusting for Elston grade, lymph-node status, PR status, tumor size, treatment, HRT use, and age. The association between expression profile and survival was validated in a separate cohort of adjuvantly treated patients with breast cancer. On a more speculative note, a comparison with cell line data indicated that long-term use of HRT may exert a tamoxifen-like effect on ER-positive breast-cancer cells. The majority of HRT users exhibiting an HRT-like gene expression pattern used a combined estrogen+progesteron regimen, however, owing to lack of power, we are unable to attach any significance to this finding.

Our results indicate that HRT use appears to alter only the expression profiles of ER-positive breast cancers. This finding is consistent with known biological and clinical observations, given that only ER-positive cells are expected to preferentially respond to estrogen exposure. In agreement with our findings, the Tamoxifen Chemoprevention Trial [31] showed reduction of breast-cancer risk only for ER-positive tumors, further supporting the fundamental specificity of the in vivo ligand-estrogen receptor effects.

In the validation cohort, the superior outcome of the HRT-associated cluster was apparent only in those patients receiving adjuvant tamoxifen, suggesting that we can identify patients who preferentially benefit from tamoxifen therapy. This observation is made more noteworthy by the fact that these patients had lower ER protein levels and would normally be considered less likely to respond to tamoxifen. Thus, the expression pattern of a tumor may add predictive value for the response to hormonal treatment to the ER protein levels.

Paradoxically, although HRT is considered an ER agonist, the expression profile induced after prolonged use of HRT was opposite to that of estradiol and consistent with tamoxifen effects (Figure 4) in MCF-7 cells. The exact ramifications of this observation are not clear, but it is known that very high doses of estradiol (e.g. diethylstilbestrol) induce clinical tumor regression, similar to anti-estrogens [32]. Intriguingly, recent experimental evidence suggests that estradiol induces apoptosis in breast-cancer cells that have developed resistance to tamoxifen therapy [33,34]. The idea that tamoxifen therapy following estradiol stimulation may have a similar effect as estrogen following tamoxifen on cell death is interesting from a therapeutic point of view, but requires further investigation.

Earlier studies have found HRT-use to be associated with lower mortality [35,36], which has in part been ascribed to the so called "healthy drug-user effect", i.e. that women using HRT are healthier than average to begin with. Previous studies of the influence of HRT on breast-cancer characteristics and prognosis have yielded somewhat conflicting results. Observational studies indicate less aggressive tumor characteristics in HRT users [5-8,37-42], while the only randomized study published to date shows that tumors of HRT users are larger and more likely to have spread outside the mammary gland [1]. Favorable tumor characteristics are expected if we assume that HRT users are under closer surveillance and thus would be diagnosed earlier in the tumor progression than non-users. Similarly, improved breast-cancer survival, shown in some studies [43-45], is also anticipated, although the specific contribution from surveillance and actual biological effects of HRT, respectively, are not readily disentangled. Jernstrom et al, however, showed that survival was better even after adjusting for stage [45], which supports the notion that HRT associated tumors are biologically different.

In line with our findings, no significant difference in recurrence or survival was seen in a case-control study when comparing 142 patients with breast cancer using HRT at time of diagnosis with 284 age-matched patients not using HRT [39]. ER levels were, however, both qualitatively and quantitatively decreased in HRT users. This finding was replicated in postmenopausal monkeys (*Cynomolgus macaques*), where nearly 3 years of exposure to HRT, including estradiol and progesterone, gave significantly lower levels of ER in breast tissue [46].

Among the genes most strongly regulated in the HRT- and non-HRT associated clusters, many were involved in either DNA repair (e.g. RRM2) or cell-cycle regulation (e.g. p63). Regulated by p53, RRM2 is essential for DNA synthesis and repair46. As a p53 homologue, the p63 gene plays a role in tumor progression and differentiation. In our study, p63 was over-expressed in HRT associated tumors and has previously been found to be exclusively expressed on the protein level in normal breast parenchyma, partially expressed in ductal hyperplasia, rarely expressed in carcinoma in situ, and not expressed in invasive carcinomas [48]. Whether patients with invasive tumors used HRT at time of diagnosis was not stated.

The non-randomized setting of our study is a major weakness. It could be argued that the ER negativity seen in HRT users was confounded by their younger age compared with non-users or by a preferential difficulty to include small ER-positive tumors from HRT users (who may be under closer surveillance and thus diagnosed with smaller tumors). Reassuringly, the association between gene-expression profile and survival remained significant after adjustment for age, stage, and grade. However, factors related to both HRT use and tumor biology, e.g. level of endogenous estrogen and receptor proteins or body weight, would still confound our results. Postmenopausal breast-cancer risk associated with high body mass index is largely the result of increase in bioavailable estradiol [49]. However, the increased risk of postmenopausal breast cancer associated with HRT seems confined to non-obese women, thus adding complexity to the relationship between the reason for taking HRT, endogenous factors, and breast-tumor biology [50]. Furthermore, the small sample size reduces precision, leaving subtle differences undetected.

We note in closing that we have not attempted to optimize the 276-gene signature for prediction through the use of cross-validation, as for example done by Pawitan et al [17]. The purpose of this study has been entirely exploratory, and we use prediction only incidentally to validate the superior outcome for the HRT-like expression profile;; the tumor subclasses may have clinical implications, but the gene signature is not intended as a clinical tool, to compare these [13].

Despite recent advances in therapy, approximately one-quarter of all women diagnosed with breast cancer will die from the disease [51]. Improved targeting of future therapies is thus of utmost importance. Our study provides for the first time a description of the effect on the tumor gene expression of HRT use.

## Conclusion

HRT alters the expression profiles of ER-positive breast cancers and patients with an HRT-related expression profile had a better prognosis despite lower ER levels. The superior prognosis seen with a HRT-associated expression pattern, also validated in a separate cohort of patients with breast cancer, remained stable even after adjusting for Elston grade, lymph node status, progesterone receptor status, tumor size, treatment, and age. The favorable survival in HRT users seemed to be confined to those treated with tamoxifen. We speculatively propose that similarities in gene expression between HRT-exposed tumors and tamoxifen-treated breast cancer cells may indicate the mechanism by which the HRT profile is related to survival.

## Competing interests

The author(s) declare they have no competing interests.

## Authors' contributions

PH co-wrote the draft and coordinated the research. AP performed the statistical analysis and co-wrote the draft. JB conceived of the study and collected the clinical information. FH collected the gene expression data for the study cohort. CYL contributed cell-line information and microbiological interpretation. ETL co-wrote the manuscript. HN provided independent pathological assessments. YP conceived of the study and contributed to the statistical analysis. PS conceived of the study and revised the original draft. LS collected the histological material. JS provided the gene-expression data for the validation cohort. SW co-wrote the draft. JO provided microbiological background. JB coordinated the research and co-wrote the draft.

## Pre-publication history

The pre-publication history for this paper can be accessed here:



## Supplementary Material

Additional file 1 **Supplementary report**. SupplementaryReport.pdf is a PDFfile containing additional information on the statistical analysis of the expression data and the comparison with the cell- line data, as well as detailed descriptions of additional data files 2 and 3. Click here for file

Additional file 2 **Annotated list of 276 HRT- related genes**. HRTrelated_GeneList.xls is an Excel spreadsheet containing the age-adjusted list of HRT-related genes, with links to EntrezGene and GenBank at NCBI. See SupplementaryReport.pdf for description. Click here for file

Additional file 3 **Annotated list of 84 genes used for cell line comparisons**. HRTrelated_FoldChanges.xls is an Excel spreadsheet containing the genes that matched between the study cohort and the cell line experiments, annotated and linked to EntrezGene. See SupplementaryReport.pdf for description. Click here for file

## Figures and Tables

**Table 1:** Characteristics of the HRT and non-HRT users in the full study cohort (n = 88) and for estrogen-receptor-positive patients (n = 72): Characteristics of the HRT and non-HRT users in the study population

	**No HRT**		**HRT**		
	**All n = 56**	***ER+ n = 50***	**All n = 32**	***ER+ n = 22***	**P value^a^**

Age at breast cancer diagnosis^b^	**67.2 **± 11.0		**60.1 **± 8.6		0.001
		***67.8 ***± *10.8*		***59.9 ***± *8.7*	*0.004*
Tumor size, mm^b^	**23.0 **± 12.4		**26.1 **± 17.1		0.39
		***23.4 ***± *12.8*		***26.2 ***± *18.4*	*0.47*
Affected lymph nodes	**40% **21/52		**39% **12/31		1.00
		***41% *** *19/46*		***43% *** *9/21*	*1.00*
Estrogen receptor-negative tumors^c^	**11% **6/56		**31% **10/32		0.02
		*N/A*		*N/A*	*N/A*
Estrogen receptor-protein, fmol/g^d^	**1.8 **± 2.1		**0.8 **± 1.5		0.01
		***2.0 ***± *2.2*		***1.2 ***± *1.8*	*0.11*
Progesterone receptor-negative tumors^c^	**27% **15/56		**41% **13/32		0.23
		***18% *** *9/50*		***23% *** *5/22*	*0.75*
Recurrence-free survival^e^	**71% **40/56		**84% **27/32		0.20
		***72% *** *36/50*		***86% *** *19/22*	*0.24*
Elston grade					0.61
					*0.27*
Grade I	**18% **9/51		**23% **7/30		-
		***20% *** *9/45*		***33% *** *7/21*	-
Grade II	**45% **23/51		**50% **15/30		-
		***49% *** *22/45*		***52% *** *11/21*	-
Grade III	**37% **19/51		**27% **8/30		-
		***31% *** *14/45*		***14% *** *3/21*	-

Values for the ER+ cohort are shown in italics. Means and percentages are shown in bold, standard deviations and counts in plain font. ^a ^Calculated via a t-test for age, size, and estrogen-receptor protein, via Fisher's exact test otherwise. ^b ^Mean plus/minus standard deviation. ^c ^Measured through an immunoassay in which monoclonal antibodies react with the receptor protein in cytosol from tumor homogenates, >0.5 fmol receptor protein/g of cytosol protein was considered as receptor positive. ^d ^The immunoassay measurement as described in ^c ^above.^e ^Measured as death or distant metastasis within 5 years of diagnosis.
