# Supplementary material for: Hormone-replacement therapy influences gene expression profiles and is associated with breast-cancer prognosis: a cohort study
Source: BMC Med. 2006 Jun 30;4:16. doi: 10.1186/1741-7015-4-16 (PMC1555602; doi:10.1186/1741-7015-4-16)
Supplement: Additional file 1 — Supplementary report. SupplementaryReport.pdf is a PDFfile containing additional information on the statistical analysis of the expression data and the comparison with the cell- line data, as well as detailed descriptions of additional data files 2 and 3. [file 1741-7015-4-16-S1.doc]

Supplementary Report April 3, 2006

**Hormone-replacement therapy influences gene expression profiles and is associated with breast-cancer prognosis**

Hall et al.

**Focus on estrogen-receptor-positive tumors**

Starting with all 88 postmenopausal patients with sufficient information on use of hormone-replacement therapy (HRT), we wanted to assess the signal related to HRT use contained in the 11,295 probe sets. For this purpose, we computed for each probe set the Welch t statistic for comparing the mean expression levels for HRT users (n=32) and non-users (n=56). The distribution of these 11,295 t statistics is shown as a solid-line curve in the top graph of supplementary Figure 1, with negative values indicating down-regulation in HRT users.

Subsequently, we computed the complete set of t statistics for 100 random arrangements of the 88 patients in groups of 32 and 56; owing to the random assignment, these t statistics are generated under the null hypothesis of no differential expression between HRT users and non-users. The distribution of these 1,129,500 t statistics is summarized by the broken-line curve in the top graph of supplementary Figure 1. A comparison shows that overall there were more large absolute t statistics observed than expected under the null hypothesis, indicating HRT related regulation among the probe sets, and that the majority of these t statistics are negative, indicating down-regulation in HRT users.

In order to confirm our initial working hypothesis that HRT regulation has stronger impact for ER-positive tumors, we repeated this (exploratory) analysis separately for ER-positive and ER-negative tumors, shown as lower left and lower right graphs in supplementary Figure 1. A comparison shows that looking at the ER-positive tumors on their own did not change the observed distribution of t statistics. For ER-negative tumors, however, the observed distribution and the distribution expected under the null hypothesis are very similar, indicating that there is little HRT-related signal in the expression values of these tumors. Based on these preliminary findings, we decided to focus the further analysis exclusively on the ER-positive tumors.

**Identification of differentially expressed genes**

We computed gene-wise test statistics t_i_ to measure the strength of the relationship between the expression of gene g_i_ and the clinical variable of interest. For the association with HRT use, we calculated the two-sample Welch t statistic; for the association with age, we used the Spearman rank correlation coefficient. Larger absolute values of either statistic correspond to a stronger association with HRT status and age, respectively.

The cut-off point for deciding that a gene g_i_ is significantly differentially expressed is not based on the usual p values. Instead, we select genes based on their local false discovery rate (FDR) as described in [1]. The quantity fdr_i_ associated with gene g_i_ specifies the proportion of false-positive results that can be expected among genes with the same test statistic t_i_. Equivalently, we can interpret 1 minus fdr_i_ as the probability that gene g_i_ is truly associated with the clinical variable of interest.

The use of FDRs for microarray expression data has become popular over recent years, as they address the problem of multiple testing for thousands of genes in parallel in a more realistic manner than the traditional control of family-wise error rates and the corresponding p value correction schemes [1-5]. Furthermore, the FDR can be computed in an entirely nonparametric manner, based on permutations of the original data, eliminating the need for specific distributional assumptions.

**Selecting HRT-related genes and eliminating age-related genes**

We identified 331 genes associated with HRT use at a local FDR of <20%. In order to correct for the fact that HRT use and age are significantly related in the study cohort (Table 1 of the paper), we eliminated all genes from this list that were significantly associated with age at a local FDR of <40%. The resulting list of 276 genes was used for the subsequent supervised and unsupervised classification, and is shown in full in the supplementary data file 1 [HRTrelated_GeneList.xls] described at the end of this report.

Note that the usual global false-discovery rate (FDR) as introduced by Benjamini and Hochberg (1995) applies to sets of genes instead of individual genes as with the local FDR. It can be directly estimated as the average local FDR of all the genes in a specific set. In our case, the collection of all 331 genes related to HRT at a local FDR of <20% has global FDR of 15%, i.e. we expect 331×0.15=~50 genes to be false positives. In the same manner, the list of 597 eliminated genes related to age at a local FDR of <40% has a global FDR of 29%, corresponding to generous 597×0.29 or approximately 173 expected false positives. 

**Unsupervised classification of the study cohort**

The 72 patients were grouped according to the expression pattern of the 276 previously selected genes. Prior to clustering, the expression values of each gene were robustly standardized by subtracting the gene-wise median and dividing by the gene-wise interquartile range. We performed hierarchical clustering based on Euclidean distances and using complete linkage.

**Assessing the robustness of the unsupervised classification**

We have used consensus clustering to assess the sensitivity of the clustering used in the analysis. We follow the procedure as outlined in [6]: variability is added to the data by repeatedly and randomly selecting subsets among the patients; these subsets are then run through the clustering procedure. At the end, we record for each pair of patients the percentage of runs where they ended up in the same cluster.

The resulting consensus matrix can then be inspected visually for signs of instability, i.e. samples that were classified differently than in the full data in a non-negligible proportion of cases. Supplementary Figures 2 to 4 show the consensus matrices when varying different parameters of the clustering procedures. In all figures, rows and columns correspond to the set of 72 samples used in the study, and the color at the intersection of a specific row and column indicates the degree of consensus between the corresponding two samples, on a scale from pure red (indicating >90% consensus) to pale yellow (indicating <10% consensus). In each subfigure, samples marked by X were classified as having HRT like expression pattern in the original clustering.

When using our approach on random data, we found that it produced homogenous orange pictures (not shown).

First, we studied the effect of leaving out different numbers of samples during the iterations, ranging from 7% (5/72) to 28% (20/72). This served to establish that our results do not depend crucially on a few extreme observations. As shown in supplementary Figure 2(a-b), we found that when leaving out five or 10 samples per iteration, samples in the HRT-like cluster remained together throughout. When leaving out 15 and 20 samples in supplementary Figure 2(c-d), the level of confusion naturally increased (more yellow, less red), but the HRT-like samples still clustered together in the majority of cases, and only one nonHRT-like case was persistently grouped with the HRT-like cases.

Subsequently, we studied the effect of cutting the dendrogram into different number of clusters during the iterations, instead of assuming that we would find consistently two clusters throughout. This serves to establish that the clusters we established in the full data set are themselves reasonably homogenous, and do not fall apart in the presence of extra noise in the data. When using three and four clusters, we found that the HRT-like cluster persisted, though the same non-HRT-like sample as above was grouped among them, see supplementary Figure 3(a-b). When increasing the number of clusters to five and 10 in supplementary Figure 3(c-d), we found that two HRT-like samples were now clustered apart from the rest of the HRT-like samples, though the other 83% (10/12) remained together.

Finally, we studied the effect of varying the mechanics of the underlying clustering algorithm. This is a fairly drastic way of assessing stability, as the very definition of clustering will depend of the choice of clustering scheme and the distance measure used, see [7] for common choices used here. We found however that we were able to extract the HRT-like cluster in all cases, except when using hierarchical clustering with single linkage (not shown). As supplementary Figure 4 shows, HRT-like samples generally stayed together, with the exception of the same two samples that were already troublesome in supplementary Figure 3.

**Supervised classification of the validation cohort**

In order to classify samples in the validation cohort, we first computed the group-wise means of the 276 genes in the HRT-like and non-HRT-like groups in the study cohort, resulting in two centroid mean vectors of length 276 for each cluster. For each sample in the validation cohort, we then computed the Euclidean distance to both centroids, and assigned the sample to the cluster with the closer centroid.

**Multivariate survival analysis**

Multivariate survival was modeled using the Cox proportional hazards model. The results are summarized in supplementary Table 1. The hazard ratios and their confidence intervals are computed from the Wald test statistics; the p value for each variable is based on the likelihood ratio test comparing the full model and the model without the tested variable, using the asymptotic χ^2^-distribution of the likelihood ratio.

**Fold change comparison with the cell line experiments**

The cell-line expression data is available from the Stanford MicroArray Database (SMD) at <http://smd.stanford.edu/index.shtml>. The data consists of five Stanford cDNA chips that were used to analyse MCF-7 cell lines that had undergone five different treatments: exposure to 10^-8^ M 17-β-estradiol for (a) 4, (b) 8 and (c) 24 hours, and exposure to (d) l and (e) 6 μM of tamoxifen for 48 hours. The treated samples were labeled with Cy5 (red) and compared with untreated control samples labeled with Cy3 (green).

Using the access options provided by the SMD, we downloaded all spots that were unflagged and had regression correlation between channels >0.6. Measurements for spots with identical clone IDs were averaged. This resulted in 25,631 unique clone IDs with normalized log_2_-fold changes between treatment and control, i.e. log_2_(R/G), where positive values correspond to up-regulation in treated samples, and negative values to down-regulation.

In order to focus on genes that are potentially estrogen-regulated, we filtered out all clones that did not fulfill any of the following criteria: (a) absolute log_2_-fold change of at least 0.2 for any of the three estrogen treatments, (b) absolute log_2_-fold change of at least 0.2 for any of the two tamoxifen treatments, and (c) reverse direction of the absolute largest fold change for the estrogen treatments and the absolute largest fold change for the tamoxifen treatments. The first two conditions ensure that a clone is only selected if it shows a minimum amount of regulation under exposure, corresponding to about 14% up-regulation or down-regulation on the raw (unlogged) scale. The third condition ensures that the clone exhibits some reverse regulation under estrogen and tamoxifen exposure, a reasonable requirement for estrogen regulation. We found that 4040 clones passed this filter.

We then matched the 4040 clones to the 276 probe sets on the age-adjusted list of HRT- associated genes, using the Locuslink ID as matching identifier. We found that 97 clones on the Stanford chip corresponded to 96 probesets on the Affymetrix chip, for a total of 84 unique Locuslink IDs.

For the cell-line data, we used the log_2_-fold changes of the 97 clones as downloaded from the SMD. For the breast cancer data, we calculated the log_2_-fold changes as

Mean log_2_ intensity of HRT users/mean log_2_ intensity of HRT non-users

For both cell-line and cancer data, we averaged the log_2_-fold changes for replicate Locuslink IDs, so that we ended up with log_2_-fold changes for HRT versus non-HRT, estradiol for different periods vs. control, and tamoxifen at different levels versus control for 84 genes. Positive values always indicate up-regulation in treated samples (HRT, estradiol, or tamoxifen).

Due to the matching with the list of HRT-associated genes, the log_2_-fold changes for HRT vs. non-HRT can be clearly grouped into up-regulated and down-regulated (down-regulation: log_2_(fc) ≤ −0.18, n=54, up-regulation: log_2_(fc) ≥0.20, n=30, see supplementary Figure 5). Figure 3 of the paper compares the distribution of log_2_-fold changes between HRT up-regulated and down-regulated genes under different experimental conditions.

The parallel regulation of gene expression in HRT users and cell lines treated with tamoxifen and the inverse regulation in cell lines treated with estrogen are robust features that remain apparent for a wide range of cutoff values for the gene filtering.

**Supplementary Data Files**

The age-adjusted list of 276 probe-sets related to HRT use in ER+ patients is given in file HRTrelated_GeneList.xls. This file contains for each probe set its Affymetrix ID, its name, and the associated local false-discovery rate and t statistic (positive t statistics correspond to up-regulation in HRT users); the probe sets are annotated with their LocusLink and GeneBank ID, which link directly to the NCBI databases; furthermore, we have added a short description, where available. We have also highlighted probe sets that have been identified as part of prognostic expression signatures in two previous publications [8, 9]. We have marked these genes as 'good' if the up-regulation is associated with improved prognosis in one of the references, and as 'bad' if their up-regulation is associated with worse prognosis. Note that the overlap with the expression signature in [8] is not surprising, as this study used an overlapping data set; no such overlap exists, however, with study [9], and it is notable that all ‘good’ genes are up-regulated and all ‘bad’ genes are down-regulated in HRT users.

The file HRTrelated_FoldChanges.xls contains the list of 84 unique genes (as identified by their LocusLink ID) that were both on the age-adjusted list of HRT related genes and were candidates for estrogen regulation in the cell-line experiments decribed above. For each gene, we list the name, the LocusLink ID (linked to NCBI’s EntrezGene), and the relevant fold changes for HRT users, three different estrogen exposures and two different tamoxifen exposures in cell lines; fold changes are given in log_2_ scale, and positive values always correspond to up-regulation in treated patients or cell lines (as opposed to untreated/controls). We have again highlighted the good/bad prognostic genes, and we have added the Affymetrix IDs for the probe sets corresponding to the LocusLink IDs (multiple probe sets for the same gene are seperated by ||).

**References**

1. Efron B, Tibshirani R, Storey JD, Tusher V: **Empirical Bayes analysis of a microarray experiment.** *J. Am. Stat. Ass.* 2001, **96:**1151-1160.
2. Liao J, Lin Y, Selvanayagam ZE, Shih WJ: **A mixture model for estimating the local false discovery rate in DNA microarray analysis.** *Bioinformatics* 2004, **20:**2694-2701.
3. Pawitan Y, Michiels S, Koscielny S., Gusnanto A. Ploner, A: **False discovery rate, sensitivity and sample size for microarray studies.** *Bioinformatics* 2005, **21:**3017-3024.
4. Reiner A, Yekutieli D, Benjamini Y: **Identifying differentially expressed genes using false discovery rate controlling procedures.** *Bioinformatics* 2003, **19:**368-375.
5. Tsai C, Hsueh H, Chen JJ: **Estimation of false discovery rates in multiple testing: application to gene microarray data.** *Biometrics* 2003, **59:**1071-1081.
6. Bourquin JP, Subramanian A, Langebrake C, Reinhardt D, Bernard O, Ballerini P, Baruchel A, Cave H, Dastugue N, Hasle H, Kaspers GL, Lessard M, Michaux L, Vyas P, van Wering E, Zwaan CM, Golub TR, Orkin SH: **Identification of distinct molecular phenotypes in acute megakaryoblastic leukemia by gene expression profiling.** *Proc Natl Acad Sci U S A* 2006, **103:**3339–3344.
7. Eisen MB, Spellman PT, Brown PO, Botstein D: **Cluster analysis and display of genome-wide expression patterns.** *Proc Natl Acad Sci U S A* 1998, **95:**14863–14868.
8. Pawitan Y, Bjohle J, Amler L, Borg AL, Egyhazi S, Hall P, Han X, Holmberg L, Huang F, Klaar S, Liu ET, Miller L, Nordgren H, Ploner A, Sandelin K, Shaw PM, Smeds J, Skoog L, Wedren S, Bergh J: **Gene expression profiling spares early breast cancer patients from adjuvant therapy: derived and validated in two population-based cohorts.** *Breast Cancer Res.* 2005, **7:**R953-R964.
9. van 't Veer LJ, Dai H, van de Vijver MJ, He YD, Hart AA, Mao M, Peterse HL, van der Kooy K, Marton MJ, Witteveen AT, Schreiber GJ, Kerkhoven RM, Roberts C, Linsley PS, Bernards R, Friend SH: **Gene Expression Profiling Predicts Clinical Outcome of Breast Cancer.** *Nature* 2002, 415:530-536.

Supplementary Table 1. Hazard ratios (HR) and 95% confidence intervals (95%CI)) from Cox proportional hazards models

|  | Study cohort (n=72) | | Validation cohort (n=131) | | Treated in validation  cohort (n=51) | | Untreated in validation  cohort (n=81) | |
| --- | --- | --- | --- | --- | --- | --- | --- | --- |
|  | HR (95%CI) | p | HR (95%CI) | p | HR (95%CI) | p | HR (95%CI) | p |
| Elston grade |  | 0.148 |  | 0.424 |  | 0.777 |  | 0.083 |
| Elston 2 vs 1 | 1.70 (0.42- 6.95) |  | 1.23 (0.66-2.30) |  | 1.34 (0.36-4.97) |  | 1.55 (0.70-3.44) |  |
| Elston 3 vs 1 | 0.31 (0.03-3.42) |  | 1.92 (0.73-5.06) |  | 1.78 (0.35-9.10) |  | 5.75 (1.45-22.87) |  |
| PGR positive | 0.17 (0.05-0.58) | 0.004 | 0.64 (0.26-1.56) | 0.337 | 1.11 (0.30-4.12) | 0.871 | 0.60 (0.15-2.34) | 0.479 |
| Age (per 10 years) | 1.63 (0.70-3.79) | 0.252 | 1.88 (1.36-2.62) | <0.001 | 1.61 (0.99-2.61) | 0.057 | 6.39 (2.99-13.68) | <0.001 |
| Size (per 10 mm) | 1.81 (0.98-3.34) | 0.054 | 1.13 (0.80-1.58) | 0.494 | 1.49 (0.91-2.47) | 0.122 | 0.76 (0.36-1.62) | 0.469 |
| Stage |  | 0.100 |  | 0.003 |  | 0.318 |  | <0.001 |
| Stage 2 vs 1 | 0.26 (0.03-2.42) |  | 0.95 (0.40-2.23) |  | 0.44 (0.04-4.38) |  | 2.28 (0.59-8.77) |  |
| Stage 3 vs 1 | 1.50 (0.30-7.60) |  | 2.61 (1.29-5.25) |  | 0.92 (0.11-7.65) |  | 1053 (64.2-17291) |  |
| HRT ongoing | 1.76 (0.32-9.70) | 0.521 |  |  |  |  |  |  |
| HRT like profile | NA^a^ | 0.001 | 0.73 (0.44-1.23) | 0.237 | 0.23 (0.08-0.62) | 0.001 | 2.65 (1.12-6.28) | 0.022 |

^a^Hazard ratio and confidence interval cannot be computed owing to lack of events (no death or regression) for the HRT-like cluster.


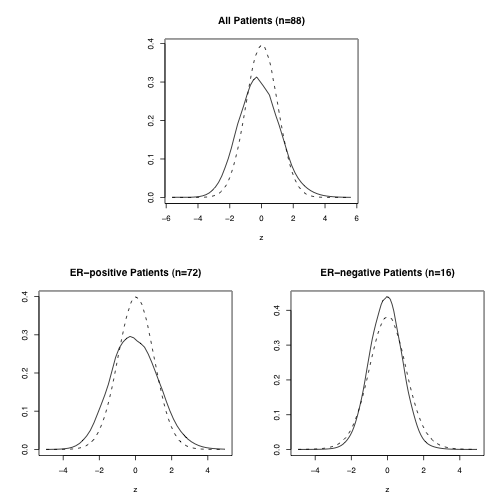


Supplementary Figure 1: Distribution of t statistics comparing HRT users and non-users. Solid lines, observed distribution; broken line,: the distribution under the null hypothesis of no differential expression between HRT users and non-users

.

*Supplementary Figure 2: Results of consensus clustering when varying the number of samples left out randomly in each iteration. Results shown are for 50 iterations, Euclidean distances, and complete linkage hierarchical clustering into two clusters. Samples classified as HRT-like in the paper are marked as X.*

*Supplementary Figure 3: Results of consensus clustering when varying the number of clusters into which samples are split in each iteration. Results shown are for 50 iterations, 10 left out samples in each iteration, Euclidean distances, and complete linkage hierarchical clustering. Samples classified as HRT-like in the paper are marked as X.*

*Supplementary Figure 4: Results of consensus clustering when varying either the clustering procedure or the distance measure between samples. Results shown are for 50 iterations, 10 excluded samples in each iteration, and by default Euclidean distances and complete linkage hierarchical clustering; the title of the subfigures indicate which clustering scheme or distance measure was substituted. Samples classified as HRT-like in the paper are marked as X.*


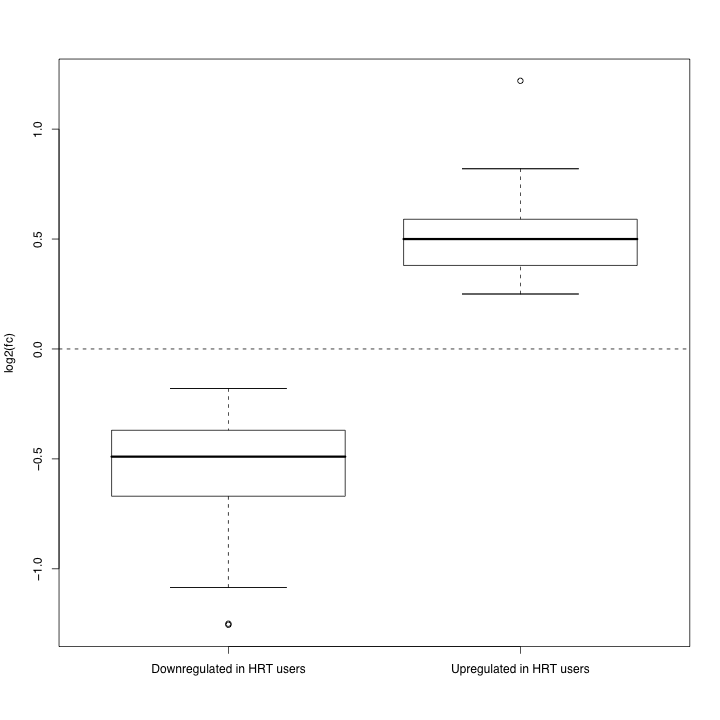


Supplementary Figure 5: Log_2_-fold changes between HRT users and non-users for 84 of the age-adjusted list of HRT-associated genes. Note the clear distinction between down-regulated- and up-regulated genes.
